# Genetic variants of *EBI3*, tumor Epstein-Barr virus, and human cytomegalovirus status in HPV-negative oral cancer

**DOI:** 10.1590/1678-7765-2025-0492

**Published:** 2026-01-26

**Authors:** Marko Simonovic, Ruzica Kozomara, Sasa Jovic, Gordana Velikic, Elizabeta Ristanovic, Nikoleta Djordjevski, Danijela Djuric-Petkovic, Srboljub Stosic, Gordana Supic

**Affiliations:** 1 Military Medical Academy Institute for Epidemiology Belgrade Serbia Military Medical Academy, Institute for Epidemiology, Belgrade, Serbia.; 2 Military Medical Academy Clinic for Maxillofacial Surgery Belgrade Serbia Military Medical Academy, Clinic for Maxillofacial Surgery, Belgrade, Serbia.; 3 Medical Academy University of Defense Medical Faculty of Military Belgrade Serbia Medical Academy, University of Defense, Medical Faculty of Military Belgrade, Serbia.; 4 University of Rochester Hajim School of Engineering Rochester NY USA University of Rochester, Hajim School of Engineering, Rochester, NY, USA.; 5 Military Medical Academy Institute for Microbiology Serbia Military Medical Academy, Institute for Microbiology, Serbia.; 6 Military Medical Academy Institute for Medical Research Belgrade Serbia Military Medical Academy, Institute for Medical Research, Belgrade, Serbia.

**Keywords:** Mouth Neoplasms, Oral Squamous Cell Carcinoma, Epstein-Barr virus, Human Cytomegalovirus, Epstein-Barr Virus induced gene 3, Single nucleotide polymorphisms

## Abstract

Oral squamous cell carcinoma (OSCC), a malignancy with high recurrence rate and poor survival, may be influenced by immune-related genetic variations and oncogenic viruses.

**Objective:**

This study evaluated the clinical relevance of *EBI3* polymorphisms, along with tumor Epstein–Barr virus (EBV) and Human Cytomegalovirus (HCMV) status, in prognostically adverse HPV-negative OSCCs.

**Methodology:**

*EBI3* (rs4740, rs4905, rs428253) genotyping was performed by qPCR in 95 HPV-negative OSCC patients and 108 age- and sex-matched controls. Tumor HPV, EBV, and HCMV status were assessed by qPCR. EBV viral load was calculated by exponential approach and a relative estimate of EBV copies per 10^5^ cells. Associations with overall survival (OS) and recurrence-free survival (RFS) were evaluated by Kaplan-Meier and Cox regression analyses.

**Results:**

EBV-positive tumors showed a significant association with increasing nodal stage (*P*=0.020). EBV viral load stratification (negative, low, high) presented a non-significant trend toward association with advanced tumor stage (*P*=0.060). Notably, *EBI3* rs428253 predicted worse OS in EBV-positive patients, whereas rs4740 and rs4905 variants were associated with advanced tumor stage (*P*=0.024 and *P*=0.018). rs4740 and rs4905 variants were inversely associated with OSCC risk in dominant and overdominant models. Analysis detected HCMV in 7.4% of tumors but was not clinically relevant.

**Conclusions:**

*EBI3* genetic variants and EBV status may have prognostic relevance in HPV-negative OSCC. EBV may interact with the host genetics to influence nodal metastases and outcomes, suggesting a potential EBV-*EBI3* axis, which warrants further investigation. Future precision oncology approaches may incorporate host and viral genetic markers to identify and stratify high-risk HPV-negative OSCC patients.

## Graphical abstract

**Figure:**
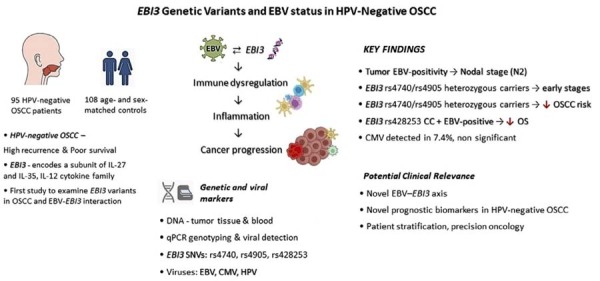
Manuscript derived from dissertation of Marko Simonovic

## Introduction

Oral squamous cell carcinoma (OSCC), the sixteenth most common malignancy worldwide, arises from the epithelial lining of the oral cavity and accounts for approximately 90% of all oral cancers. Despite advances in diagnosis and treatment, the 5-year survival rate remains poor.^1^ Besides well-known etiological factors like smoking and alcohol consumption, there is growing interest in the role of viral infections with Human papillomavirus (HPV)^2^ and Epstein-Barr virus (EBV) in head and neck squamous cell carcinoma (HNSCC)^3^ and OSCC.^4^ The HPV-negative status of oropharyngeal squamous cell carcinoma (OPSCC) is associated with a worse prognosis and poor response to treatment compared with HPV-positive counterparts, which has led to OPSCC classification according to HPV status and the introduction of a distinct staging system.^5^ Despite being distinct subsets of HNSCC, both OPSCC and OSCC share common risk factors such as exposure to alcohol and tobacco. HPV-negative OPSCCs are predominantly genetically driven, characterized by genomic instability, higher mutational burden, and radio- and chemotherapy resistance, resulting in poor survival. In turn, HPV-positive OPSCC usually show a survival advantage and increased responsiveness to treatment, probably due to the ability of HPV oncoproteins (E6 and E7) to elicit robust host antitumor immune responses, whereas apoptotic pathways are usually unaffected by genetic mutations.^5^Although the prognostic significance of HPV status in OSCC remains controversial, several studies have associated HPV infection with improved survival of OSCC patients,^6,7^ particularly in cases with recurrent or progressive disease.^8^ Epstein-Barr virus is a widespread human herpesvirus that infects approximately 90% of the global population, with most individuals remaining asymptomatic. While EBV is typically transmitted via saliva during childhood or adolescence,^9^ some evidence suggests potential sexual transmission routes.^10^ Reactivation may occur in immunosuppressed or immunocompromised individuals and cancer patients. EBV employs multiple immune evasion strategies, including downregulating antigen presentation, altering cytokine signaling, and expressing viral homologs of immune regulators which allows infected cells to evade detection by cytotoxic T-cells and maintain persistent inflammation in the tumor microenvironment.^11^ EBV persists in host cells throughout life largely due to the Latent Membrane Proteins (LMPs) family, which are essential for maintaining viral latency and modulating the host immune response. This virus also exerts oncogenic potential, predominantly associated with lymphoid or epithelial malignancies, including HNSCC and nasopharyngeal carcinoma (NPC), although data regarding its role in OSCC development or progression remains inconclusive, particularly in the HPV-negative subtype.^3,12^ Other herpesviruses such as Human Cytomegalovirus (HCMV) have not been clearly linked to HNSCC, but a recent study suggested an oncoprotective effect in OSCC and OPSCC.^13^

A growing body of evidence suggests that the immunological landscape, antitumor immune response, and tumor progression are significantly influenced by genetic variations in immune-related genes in both tumor and immune cells. EBV-induced gene 3 (*EBI3*), originally identified as a gene induced in human B lymphocytes following EBV infection, belongs to the interleukin-12 (IL-12) cytokine family and exhibits important immunomodulatory functions. *EBI3*, located on chromosome 19p13.3, encodes a p40 subunit that forms heterodimers with p28, p35, and p19 subunits to generate distinct cytokines — IL-27, IL-35, and IL-39, respectively.^14^ *EBI3* expression is induced during inflammatory responses and is implicated in infectious diseases, autoimmunity, and various malignancies.^14^ As a component of these heterodimeric cytokines, *EBI3* might contribute to diverse roles in carcinogenesis, showing context-dependent expression patterns across tumor types.^15^

Genetic variants in the *EBI3* gene may affect its stability, structure, or function, thereby modifying its involvement in inflammatory pathways and potentially contributing to cancer progression or susceptibility.^22^ *EBI3* rs4740 is a nonsynonymous single nucleotide polymorphism (SNP) in exon 5 where a G-to-A substitution results in the valine to isoleucine change at position 201 within the fibronectin type III domain of *EBI3*.^22^ rs4905 polymorphism is a synonymous variant in exon 5, representing an A/G substitution, whereas rs428253 is located in intron 1 and involves a C/G substitution.^23,24^

The intersection of viral infection, host immunogenetics, and tumor biology represents a little explored paradigm in oral cancer research. While *EBI3* variants have been studied in various cancers and linked to immune dysfunction, their potential effects in the context of EBV infection remain unexplored. Understanding these interactions could reveal novel biomarkers for patient stratification and therapeutic targeting.

Thus, this study investigated the associations of *EBI3* (rs4740, rs4905, and rs428253) genetic variants with clinicopathological features, oral cancer risk, overall and progression-free survival, and tumor EBV and HCMV status in HPV-negative OSCC patients.

## Methodology

### Study patients

The study group consisted of 95 patients diagnosed with OSCC and treated at the Clinic for Maxillofacial Surgery, Military Medical Academy, Belgrade, between 2014 and 2023. All patients were between 18 and 80 years old, treatment-naïve prior to tumor resection, and had operable, histologically confirmed OSCC. Primary surgical resection was followed by adjuvant radiotherapy or chemoradiotherapy according to tumor stage and nodal involvement. Only patients with HPV-negative tumors were included in the study. Patients with HPV-positive tumors, inoperable disease, and those who had previously received oncological or systemic immunosuppressive therapy were excluded. The control group consisted of 108 healthy individuals with no prior history of malignancies, chronic infections, or autoimmune disorders, who were age- and sex-matched with the OSCC cohort. All participants were Caucasians of Serbian origin. The study was approved by the Ethics Committee of the Military Medical Academy, Belgrade (approval number 1494-6), and the Ethics Committee of the Medical Faculty of the Military Medical Academy, Belgrade (approval number 52/2024). All participants provided informed consent, and confidentiality and data protection standards were strictly followed. All procedures involving human tissue samples adhered to the ethical standards in accordance with the 1964 Helsinki Declaration and its later amendments.

### DNA extraction and genotyping

Tumor tissue was collected immediately after surgical excision, then frozen and stored at -40°C until DNA extraction. DNA was isolated from tumor samples using TRIzol reagent (Invitrogen, Waltham, Massachusetts, USA) according to the manufacturer’s instructions and stored at -40^o^C until further use. Genomic DNA from the blood of healthy controls was extracted using the commercial ExtractMe kit (Blirt, Gdansk, Poland). DNA quality was assessed spectrophotometrically and by agarose gel electrophoresis to ensure suitability for downstream qPCR analysis. Additionally, β-globin amplification was used as an endogenous control to confirm DNA quality and normalize viral quantification.

All genetic variant analyses were conducted in both OSCC cases and controls to assess cancer risk. The selected polymorphisms in the *EBI3* (rs4740, rs4905, and rs428253) gene were genotyped by real-time PCR using the allelic discrimination method and TaqMan SNP Genotyping Assays (Applied Biosystems, Waltham, Massachusetts, USA) on the QuantStudio 5 Real-Time PCR System (Applied Biosystems, Waltham, Massachusetts, USA).

### OSCC cohort tumor HPV, EBV, and HCMV status and EBV viral load quantification

Since blood analysis is more likely to identify past exposures than viral presence in the malignant microenvironment, tumor tissue analysis was chosen to specifically assess viral DNA. Detection of viral DNA in blood samples from controls would indicate prior exposure to these ubiquitous viruses in otherwise healthy individuals. Total DNA extracted from the tumor tissue of OSCC patients was analyzed for viral status using commercial qPCR kits. Tumor HPV status was assessed by the AmpliSens HPV HCR screen-titre-14-FRT PCR kit (Moscow, Russia). EBV and HCMV were assessed by the commercial Sacace Biotechnologies kits (CE-IVD certified), specific for EBV (LMP gene) (Cat. #V9-100FRT), and HCMV (MIE gene) (Cat. #V7-100/2FRT), following the manufacturers’ instructions. The assays target conserved regions of the viral genome, using pre-validated primers and TaqMan probes which are proprietary to the manufacturer. These tests allow qualitative and quantitative detection of viral DNA. Instead of specifically amplifying Latent Membrane Protein LMP1 or LMP2 isoforms, the Sacace EBV Real-TM Quant kit amplifies a latent membrane protein (LMP) region in general. Viral load quantification of EBV DNA in tumor tissues was performed using the comparative cycle threshold (Ct) method. Ct values for EBV and the endogenous control gene (β-globin) were used to calculate the difference in Ct (ΔCt), defined as ΔCt = Ct_target (EBV) – Ct_reference (β-globin). Due to the exponential nature of qPCR amplification, the viral load was calculated according to an exponential approach and a relative estimate of EBV copies per 10^5^ cells, defined as 2^(–Ct)^ x 200,000, in which 200,000 corresponds to the estimated number of diploid genome equivalents per 10^5^ cells. The log_10_ transformation was also used to provide a more normalized distribution of the wide variability of EBV levels for subsequent statistical analyses and correlation. For categorical analyses and association with clinical parameters and survival, tumors were classified as EBV-negative (no amplification), EBV-low, or EBV-high, and the median value was used as the cutoff for EBV-low *versus* EBV-high groups.

### Bioinformatics analysis of the investigated polymorphisms

The HaploReg v4.2^25^ (https://pubs.broadinstitute.org/mammals/haploreg/haploreg.php) and Ensembl (https://www.ensembl.org/index.html) platforms were used for the initial selection of genetic variants and to predict their regulatory potential. The Allele Frequency Aggregator (ALFA) database (www.ncbi.nlm.nih.gov/snp/docs/gsr/alfa/) was assessed to compare allele and genotype frequencies of the selected SNPs with population-level reference data from NCBI’s Database of Genotypes and Phenotypes (dbGaP). The RegulomeDB platform (https://regulomedb.org), which integrates data from ENCODE and other projects, was used to evaluate the functional relevance of the investigated polymorphisms. Additionally, ChIP-seq data from RegulomeDB were explored to assess potential overlap with transcription factor binding sites.

### Statistical analysis

Statistical analyses were performed using SPSS version 20 (IBM SPSS Statistics version 20). Associations between the categorical variables were assessed using the χ^2^ test or Fisher’s exact test when appropriate. Overall survival (OS) and recurrence-free survival (RFS) were estimated using the Kaplan–Meier method and the log-rank test. Recurrence-free survival was defined as the time from diagnosis to the first evidence of tumor recurrence or last follow-up and overall survival was defined as the time from surgery until death from any cause or last follow-up. Gene-virus interaction was analyzed by stratified survival according to EBV status. Hazard ratios (HR) with 95% confidence intervals (CI) were estimated using Cox proportional hazards regression analysis.

Logistic regression analysis, adjusted for sex and age, tested the association between the analyzed polymorphisms and OSCC risk, providing odds ratios (ORs) and 95% CIs. Genetic associations were evaluated under additive, dominant, recessive, over-dominant, and genotypic inheritance models. Due to the hypothesis-driven selection of variants, their limited number, and the lack of transcriptomic data, correction for multiple testing was not applied. Two-sided *P*-values below 0.05 were considered statistically significant.

## Results

### *EBI3* rs4740, rs4904, and rs428253 polymorphisms and their potential impact by bioinformatics prediction

According to HaploReg v4.2^25^ and ENSEMBL, the polymorphisms in *EBI3* rs4740, rs4905, and rs428253 may result in enhancer histone marks and are located in a region with DNase I hypersensitivity (Table 1). *EBI3* polymorphisms rs4740 and rs4905 show strong linkage disequilibrium (r’=1 and D’=1). The rs4740 variant affects several motifs, including BDP1 (bromodomain-containing protein), whereas rs4905 alters Sox motifs (Sry-related HMG-box proteins), and rs428253 influences multiple motif changes, including TATA and HDAC2. Moreover, rs428253 lies within a binding site of the BATF transcription factor (Basic Leucine Zipper ATF-like Transcription Factor) and exhibits histone modification changes. RegulomeDB indicates high regulatory potential for *EBI3* rs428253 (score: 0.95) and moderate regulatory potential for rs4740 and rs4905 (score: 0.55). ChIP-seq data show that *EBI3* rs4740 and rs4905 predominantly occupy polycomb-repressed regions in various tissues, including epithelial tissues, and that these variants disrupt multiple transcription factor binding motifs, including POLR2A (RNA polymerase II subunit) and CEBPA (CCAAT/Enhancer Binding Protein Alpha). Notably, rs428253 overlaps with IRF1 (Interferon Regulatory Factor 1) transcription factor binding sites across different immune and non-immune cells, suggesting potential immune regulation.

**Table 1:** The characteristics of genotyped polymorphisms and their potential impact prediction.

Gene	Location	rs number	SNP Change, Major/Minor allele	Varian type, Region	Amino Acid Change	HaploReg v4.1 prediction of motif changes	
						Enhancer histones marks	Motifs changed
*EBI3*	19p13.3	rs4740	G/A	exon 5, missense	Val201Ile, V201I	+	BDP1
							Nkx-2
*EBI3*	19p13.3	rs4905	A/G	exon 5, synonymous		+	Sox
							THAP1
*EBI3*	19p13.3	rs428253	G/C	intron 1		+	AIRE, DMRT7, HDAC2, HNF1, Irf, SEF-1, TATA
							TATA

BDP1 (bromodomain-containing protein). Nkx-2 (homeobox transcription factor). Sox (Sry-related HMG box). THAP1 (THAP domain containing 1). AIRE (Autoimmune Regulator). DMRT7 (Doublesex and Mab-3 Related Transcription Factor). HDAC2 (Histone Deacetylase 2). HNF1 (Hepatocyte Nuclear Factor 1). Irf (Interferon Regulatory Factor). SEF-1 (Serum Response Factor-like Element).

### Association between gene polymorphisms, EBV, and HCMV infection with demographic and clinicopathological features in the HPV-negative OSCC cohort

The study cohort included 95 HPV-negative OSCC patients, predominantly male (80%) and with median age of 58 years (range: 39–80) (Table 2). Most OSCC patients were current smokers (71.58%), with 18.95% non-smokers and 9.47% former smokers. Regarding alcohol consumption, 54.74% were non-drinkers, 26.32% drank moderately, and 18.95% were heavy drinkers. According to AJCC staging, 69.47% were in advanced stages (III/IV), and during follow-up, 60% of patients relapsed.

**Table 2:** Demographics and clinicopathological characteristics of the studied OSCC cohort.

Variable		Total OSCC N=95	Tumor EBV status		Tumor EBV viral load*			Tumor HCMV status	
			Negative	Positive	Negative	Low	High	Negative	Positive
			**N=56**	**N=39**	**N=56**	**N=20**	**N=19**	**N=88**	**N=7**
Sex	Male	76	44	32	44	18	14	70	6
	Female	19	12	7	12	2	5	18	1
		P	0.797		0.408			1.000	
Age (median)	58<	43	21	2	22	10	11	40	3
	≥58	52	35	17	34	10	8	48	4
		P	0.094		0.331			1.000	
Smoking	No	18	6	12	6	8	4	18	0
	Yes	68	43	25	43	12	13	63	5
	Former	9	7	2	7	0	2	7	2
		P	0.035		0.044			0.114	
Alcohol	No	52	30	22	30	13	9	50	2
	Moderate	25	16	9	17	2	6	21	4
	High	18	10	8	9	5	4	17	1
		P	0.826		0.429			0.153	
Histological grade	1	65	32	33	32	17	16	53	2
	2	16	12	4	12	2	2	17	3
	3	14	12	2	12	1	1	18	2
		P	0.015		0.078			0.175	
Nuclear grade	1	55	30	25	30	12	13	53	2
	2	20	12	8	12	6	2	17	3
	3	20	14	6	14	2	4	18	2
		P	0.482		0.420			0.220	
Tumor size	T 1/2	65	41	24	41	15	9	60	5
	T 3/4	30	15	15	15	5	10	28	2
		P	0.266		0.087			1.000	
Nodal status	0	34	16	18	16	10	8	31	3
	1	50	36	14	36	7	7	46	4
	2	11	4	7	4	3	4	11	0
		P	0.020		0.081			0.604	
Stage	I/II	29	16	13	16	10	3	26	3
	III/IV	66	40	26	40	10	16	62	4
		P	0.656		0.060			0.433	
Recurrence	Negative	38	22	16	21	10	7	36	2
	Positive	57	34	23	35	10	12	52	5
		P	1.000		0.589			0.698	

* EBV viral load categories were defined as negative, low (below the median), and high (above the median), based on values calculated as 2^(–ΔCt)^ x 200,000/10⁵ cells.

Analysis detected EBV DNA in 39 out of 95 OSCC tumor samples (41%), and HCMV DNA in 7 out of 95 OSCC tumor samples (7.4%) (Table 2).The association found between tumor EBV status and nodal involvement (*P=*0.020) was statistically significant. Distribution was relatively balanced among OSCC patients without nodal involvement (N0), with 47.1% of EBV-negative cases (16/34) vs. 52.9% of EBV-positive cases (18/34). Conversely, EBV-positive patients exhibited a higher frequency of nodal metastases, with the most pronounced differences observed between the N1 and N2 subgroups. The N1 subgroup presented a marked shift, with EBV negativity detected in 72.0% of patients (36/50) compared with 28.0% of EBV-positive cases (14/50). N2 subgroup showed an increasing EBV prevalence with advancing nodal stage, where EBV positivity was detected in 63.6% of patients (7/11) whereas 36.4% of patients (4/11) had EBV-negative tumors. In contrast, the binary comparison of node-positive (N+) versus node-negative (N−) cases revealed only a trend toward significance (*P=*0.087). Additionally, EBV status was significantly associated with smoking (*P=*0.035), histological grade (*P=*0.015), and nodal status (*P=*0.020).

Median EBV viral load was 58.83 copies/10⁵ cells (range: 1.75–6,794). After log_10_ transformation, distribution was normalized with a median of 1.77 log_10_ copies/10⁵ cells (range: 0.24–3.83). EBV viral load quantification revealed no significant associations when dichotomized as high vs. low/negative. However, when stratified into three groups (negative, low, high), EBV viral load showed a tendency towards association with tumor stage (*P*=0.060), nodal status (*P*=0.081), and tumor size (*P*=0.087), albeit not statistically significant (Table 2). EBV viral load did not correlate with recurrence, age, sex, or alcohol use. No significant associations were observed between HCMV positivity and clinicopathological parameters or the genetic variants analyzed.

Table 3 provides data on the association of analyzed genetic variants with demographic and clinicopathological variables. Among *EBI3* SNPs, rs4740 and rs4905 were significantly associated with tumor stage (*P=*0.024 and *P=*0.018, respectively), whereas rs428253 was associated with histological grade (*P=*0.001). Moreover, a significant association was observed between rs4740 genotypes and EBV viral load levels (*P*=0.047) (Table 3).

**Table 3:** Association of gene polymorphisms with demographic features .

Variables	Total		*EBI3*	*EBI3*	*EBI3*
	N=95		rs4740	rs4905	rs428253
			GG/GA/AA	AA/AG/GG	GG/GC/CC
Sex	Male	76	42/19/15	41/23/12	43/30/3
	Female	19	7/9/3	7/10/2	10/8/1
	P		0.157	0.187	0.936
Age	58<	43	21/12/10	21/14/8	23/18/2
	>58	52	28/16/8	27/19/6	30/20/2
	P		0.622	0.622	0.914
Smoking	Never	18	5/8/5	5/9/4	7/10/1
	Current	68	39/19/10	38/21/9	40/25/3
	Former	9	5/1/3	5/3/1	6/3/0
	P		0.122	0.326	0.549
Alcohol	No	52	24/18/10	24/22/6	28/23/1
	Low	25	14/6/5	13/7/5	14/8/3
	High	18	11/4/3	11/4/3	11/7/0
	P		0.769	0.481	0.217
Tumor EBV status	Negative	56	31/12/13	30/17/9	34/19/3
	Positive	39	18/16/5	18/16/5	19/19/1
	P		0.096	0.558	0.320
Tumor EBV viral load*	Negative	56	30/12/14	29/17/10	33/19/4
	Low	20	8/11/1	8/11/1	10/10/0
	High	19	11/5/3	11/5/3	10/9/0
	P		0.047	0.249	0.365
Tumor HCMV status	Negative	88	47/25/16	46/30/12	51/33/4
	Positive	7	2/3/2	2/3/2	2/5/0
	P		0.448	0.397	0.203
Histological grade	1	65	29/23/13	29/27/9	32/32/1
	2	16	11/3/2	11/3/2	13/0/3
	3	14	9/2/3	8/3/3	8/6/0
	P		0.299	0.298	0.001
Nuclear grade	1	55	29/19/10	25/23/7	30/24/1
	2	20	11/6/3	11/6/3	11/7/2
	3	20	12/3/5	12/4/4	12/7/1
	P		0.560	0.491	0.578
Tumor size	1/2	65	35/20/10	34/23/8	39/25/1
	3/4	30	14/8/8	14/10/6	14/13/3
	P		0.427	0.613	0.118
Nodal status	0	34	15/15/4	14/17/3	18/15/1
	1	50	28/9/13	28/12/10	29/18/3
	2	11	6/4/1	6/4/1	6/5/0
	P		0.082	0.144	0.827
Tumor stage	I/II	29	12/14/3	11/16/2	15/13/1
	III/I	66	37/14/15	37/17/12	38/25/3
	P		0.024	0.018	0.809
Recurrence	0	38	19/13/6	18/15/5	24/13/1
	1	57	30/15/12	30/18/9	29/25/3
	P		0.655	0.726	0.467

* EBV viral load categories were defined as negative, low (below the median), and high (above the median), based on values calculated as 2^(–ΔCt)^ x 200,000/10⁵ cells.

No significant correlations were observed between EBV viral load (expressed as log_10_ copies/10⁵ cells) and *EBI3* genotypes. Specifically, Spearman correlation coefficients were ρ=0.006 (*P*=0.972) for rs4740, ρ=0.006 (*P*=0.972) for rs4905, and ρ=0.062 (*P*=0.710) for rs428253.

### Association of gene polymorphisms and EBV tumor status with overall and recurrence-free survival

**Figure 1:**
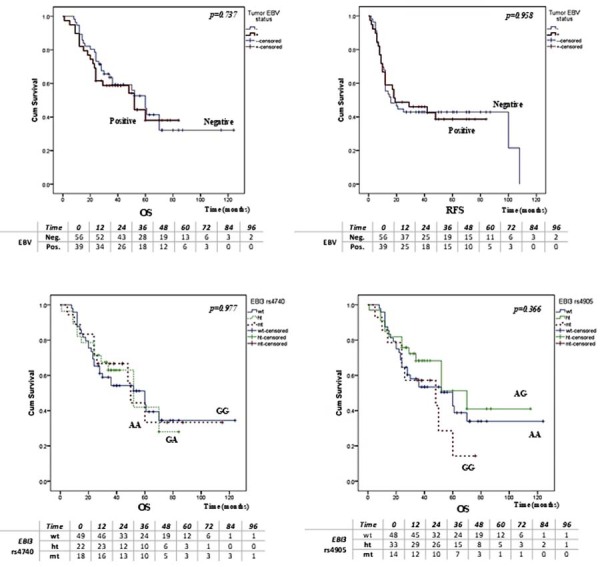
Tumor EBV status, *EBI3* rs4740, and rs4905 variants and their relationship to HPV-negative OSCC patients’ survival. The number of patients at risk at specific time points for each genotype or EBV status are presented beneath survival curves. (A) Association of tumor EBV status with OS. (B) Association of tumor EBV status with RFS. (C) Association of the EBI3 rs4740 genetic variant with OS. (D) Association of the *EBI3* rs4905 genetic variant with OS.

**Figure 2:**
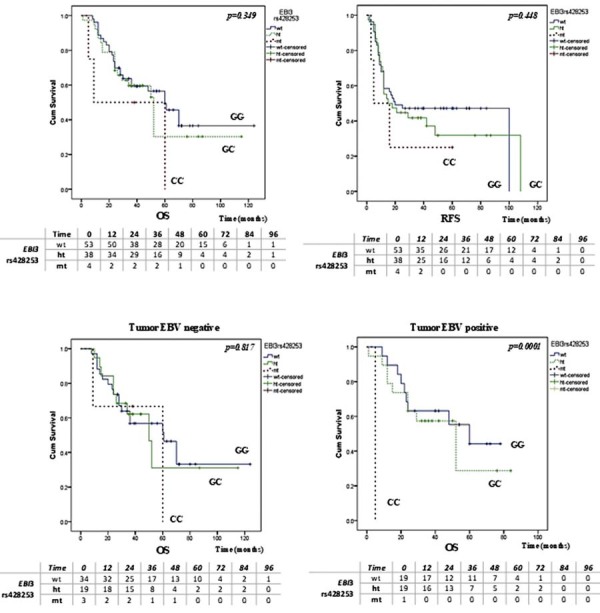
Association of the EBI rs428253 genetic variant with survival in HPV-negative OSCC patients and stratified analysis by EBV status. The number of patients at risk at specific time points for each genotype or stratified by EBV status are presented beneath survival curves. (A) Association of the rs428253 genetic variant with OS. (B) Association of the rs428253 genetic variant with RFS. (C) Association of the rs428253 genetic variant with OS in EBV-negative patients. (D) Association of the rs428253 genetic variant with OS in EBV-positive patients.

Kaplan–Meier analysis showed no significant association between EBV status and overall survival (OS) or recurrence-free survival (RFS) (*P=*0.737 and *P=*0.958, respectively) (Figure 1). The *EBI3* rs428253 variant was not associated with OS (*P=*0.349) or RFS in our study (*P=*0.448). However, stratified by EBV status, *EBI3* rs428253 was significantly associated with OS in EBV-positive patients (*P=*0.0001), but not in EBV-negative patients (*P=*0.817) (Figure 2). In EBV-positive patients, carriers of the CC genotype from the *EBI3* rs428253 variant had a significantly shorter OS than those with the GG and GC genotypes. The other two *EBI3* variants, rs4740 and rs4905, were not associated with OS or RFS of OSCC patients (Figure 1). Figures 1 and 2 present the number of patients at risk at specific time points for each genotype or EBV status are presented beneath the survival curves. HCMV positivity did not significantly associate with OS (*P=*0.539) or RFS (*P=*0.422).

The number at risk indicates patients alive and uncensored at the beginning of each time interval. Censored cases are excluded from subsequent time points. OS, overall survival; RFS, recurrence-free survival.

The number at risk indicates patients alive and uncensored at the beginning of each time interval. Censored cases are excluded from subsequent time points. OS, overall survival; RFS, recurrence-free survival.

### Cox proportional hazards regression analysis

Cox logistic regression analysis of relevant prognostic factors evaluated potential OS and RFS predictors in OSCC patients (Table 4). For OS, Cox univariate logistic regression analysis revealed that nodal metastasis increases the mortality risk over twofold (HR=2.346, *P=*0.013), whereas advanced tumor stage (III and IV) was associated with a 1.5-fold increased mortality compared with early-stage tumors (I and II) (HR=1.565, *P=*0.002). Tumor recurrence was the strongest predictor of poor prognosis by increasing the risk of death over eightfold (HR=8.560, *P=*0.0001). Multivariate analysis identified tumor recurrence (HR=7.996, *P=*0.00001) and advanced stage (HR=2.590, *P=*0.0001) as independent predictors of OS. Advanced tumor stage was an independent predictor of recurrence risk (HR=1.812, *P=*0.001).

**Table 4:** Analysis of different prognostic factors with overall survival (OS) and recurrence-free survival (RFS), according to Cox proportional hazards regression analysis.

Cox Regression Analysis	Variables		Overall survival			Recurrence-Free survival		
			HR	(95% CI)	*P*	HR	(95% CI)	*P*
Univariate Analysis	Sex		0.604	[0.271-1.347]	0.218	0.673	[0.338-1.337]	0.258
	Age		1.009	[0.977-1.042]	0.579	1.819	[1.051-3.148]	0.033
	Smoking		1.527	[0.896-2.603]	0.120	1.224	[0.745-2.011]	0.426
	Alcohol		1.533	[1.090-2.156]	0.014	1.121	[0.795-1.580]	0.515
	Tumor EBV status		1.101	[0.622-1.947]	0.742	1.014	[0.593-1.733]	0.959
	EBV viral load*		1.041	[0.737-1.472]	0.818	0.924	[0.704-1.375]	0.984
	Tumor HCMV status		1.369	[0.492-3.814]	0.548	1.441	[0.574-3.617]	0.437
	Histological grade		1.305	[0.908-1.875]	0.150	1.348	[0.947-1.920]	0.097
	Nuclear grade		1.185	[0.847-1.657]	0.321	1.525	[1.113-2.088]	0.009
	Tumor size		1.315	[1.089-1589]	0.004	1.151	[0.960-1.379]	0.128
	Nodal status		2.346	[1.199-4.594]	0.013	1.936	[1.082-3.466]	0.026
	Stage		1.565	[1.177-2.082]	0.002	1.373	[1.101-1.,712]	0.005
	Recurrence		8.560	[3.782-19.37]	0.000	-	-	-
	*EBI3*	ht vs. wt	0.948	[0,488-1.843]	0.875	0.866	[0.465-1.613]	0.650
	rs4740	mt vs. wt	0.933	[0.438-1.987]	0.858	1.045	[0.522-2.091]	0.901
	*EBI3*	ht vs. wt	0.742	[0382-1.440]	0.378	0.763	[0.421-1.385]	0.374
	rs4905	mt vs. wt	1.344	[0.629-2.871]	0.446	1.248	[0.592-2.633]	0.561
	*EBI3*	ht vs. wt	1.171	[0.647-2.119]	0.602	1.180	[0.687-2.029]	0.549
	rs428253	mt vs. wt	2.312	[0.698-7.657]	0.170	2.026	[0.616-6.662]	0.245
Multivariate analysis	Stage		2.590	[1.622-4.137]	0.0001	1.812	[1.281-2.564]	0.001
	Recurrence		7.996	[3.49-18.327]	0.00001	-	-	-

HR - Hazard ratio. Confidence interval.

* EBV viral load categories were defined as negative, low (below the median), and high (above the median), based on values calculated as 2^(–ΔCt)^ x 200,000/10⁵ cells.

### Association of analyzed genes’ polymorphisms and OSCC risk

Alongside 95 OSCC patients, 108 healthy individuals were included in the study as a control group. Most participants were male (68/108, 63%), with a median age of 58.3 years (range: 24–80), and selected to match the OSCC cohort by sex and age to minimize potential confounding effects. As shown in Table 5, a significant difference in genotype distribution was observed between OSCC patients and the age- and sex-matched control group for the *EBI3* rs4740 and rs4905 variants. *EBI3* rs4740 GA genotype showed a protective effect under the over-dominant model (OR=0.553, *P=*0.047). For the *EBI3* rs4905, the AA genotype under the dominant model was associated with an increased OSCC risk (OR=1.813, *P=*0.039), whereas the AG genotype under the over-dominant model had a protective effect (OR=0.412, *P=*0.002). The dominant model for rs4905 showed a significant increase in OSCC risk (OR=1.813, 95% CI=1.032–3.187, *P=*0.039). No differences in genotype frequencies between OSCC patients and controls were identified for the analyzed genetic polymorphism in *EBI3* rs428253.

**Table 5:** Association of analyzed gene polymorphisms and OSCC risk.

Gene/SNP	Genotype	Controls		OSCC		Adjusted OR*, [95 CI]	*P*
		N=108	%	N=95	%		
*EBI3* rs4740	GG	49	45.37	49	51.58	1	Ref.
	GA	47	53.52	28	29.47	0.609 [0.329-1.129]	0.115
	AA	12	11.11	18	18.95	1.517 [0.658-3.497]	0.328
	Additive model					1.043 [0.710-1.534]	0.829
	Recessive model - mt. vs wt+ht (Ref.)					1.877 [0.849-4.150]	0.150
	Dominant model wt vs het+mut (Ref.)					1.258 [0.721-2.195]	0.419
	Over-dominant model het vs wt+mut (Ref.)					0.553 [0.308-0.992]	0.047
*EBI3* rs4905	AA	39	36.11	48	50.53	1	Ref.
	AG	61	56.48	33	34.74	0.440 [0.241-0.801]	0.007
	GG	8	7.41	14	14.74	1.400 [0.531-3.689]	0.496
	Additive model					0.844 [0.554-1.287]	0.432
	Recessive model - mt. vs wt+ht (Ref.)					2.128 [0.849-5.336]	0.107
	Dominant model wt vs het+mut (Ref.)					1.813 [1.032-3.187]	0.039
	Over-dominant model het vs wt+mut (Ref.)					0.412 [0.233-0.728]	0.002
*EBI3* rs428253	GG	69	63.89	53	55.79	1	Ref.
	GC	36	33.33	38	40.00	1.341 [0.749-2.402]	0.324
	CC	3	2.78	4	4.21	1.842 [0.391-8.686]	0.440
	Additive model					1.346 [0.821-2.209]	0.239
	Recessive model - mt. vs wt+ht (Ref.)					1.657 [0.356-7.706]	0.520
	Dominant model – wt vs het+mut (Ref.)					0.725 [0.412-1.278]	0.266
	Over-dominant model – het vs wt+mut (Ref.)					1.299 [0.730-2.312]	0.374

*Adjusted for age and sex. CI - Confidence interval. Ref - Reference.

Our preliminary power analysis indicated that enrolling 80–90 patients with a 1:1 ratio of controls would be sufficient to achieve 80% statistical power for cancer risk analysis. Post-hoc power calculations confirmed that with 95 OSCC patients and 108 controls, the study had adequate power (>80%) to detect moderate-to-strong genetic effects on cancer risk, as observed for rs4905 in the dominant and over-dominant models. However, smaller effects were observed for rs4740 and rs428253, with estimated power of 55–60%. Sensitivity was further constrained for rs428253 by the low frequency of CC genotype (2.8% in controls, 4.2% in the OSCC cohort).

## Discussion

Oral squamous cell carcinoma (OSCC) constitutes a biologically aggressive malignancy characterized by high recurrence rates and poor survival outcomes, highlighting the urgent need for novel biomarkers to improve diagnosis, monitor disease progression, and therapeutic stratification. Recent research underscores the critical role of alterations in immune-related genes, both in tumor cells and the immune microenvironment, significantly shaping the immunological landscape, the antitumor immune response, and influencing tumor progression. The *EBI3* gene, encoding for the p40 subunit—a shared component of several cytokines in the IL-12 cytokine family—, has also drawn attention due to its various immunoregulatory and tumor-promoting roles. *EBI3* is overexpressed in multiple cancer types, including nasopharyngeal carcinoma, and contributes to tumor-associated inflammation and immune escape.^14^ Moreover, we have a growing interest in the interplay between host genomic variants and viral infections in malignant transformation and progression, particularly involving HPV and EBV, whose role in OSCC remains unelucidated and often controversial.^8-10,26,27^ Given that EBV infects over 90% of adults worldwide and that blood detections would probably indicate prior exposures, our goal was to investigate viral presence in tumor tissue for a better understanding of their role in the tumor microenvironment.

Our study provides the first report of *EBI3* genetic variants (rs4740, rs4905, rs428253) and their interaction with tumor EBV status in HPV-negative OSCC. Our findings indicate that EBV positivity is associated with clinically relevant features of HPV-negative OSCC, particularly nodal dissemination, higher histological grade, and smoking. *EBI3* rs4740 and rs4905 showed significant association with advanced tumor stage in our OSCC cohort, whereas rs428253 was linked to higher histological grade and tumor size. Notably, even though EBV status and *EBI3* variants did not significantly affect survival, our research provides novel results that rs428253 stratification had a significant prognostic impact, specifically in EBV-positive OSCC cases. These findings suggest a possible interplay between host immunogenetic background and EBV-mediated tumor progression in HPV-negative OSCC.

We observed that EBV-positive tumor status was associated with increasing nodal stage (N0–N2), suggesting a stepwise contribution of EBV to lymphatic dissemination rather than a simple node-positive/node-negative dichotomy. Even though lymph node metastasis is a common HNSCC feature, the N1 category often shows variation, exhibiting both up-staged and down-staged cases,^28^ indicating dynamic immune-tumor interactions at early nodal dissemination. In the tumor microenvironment, EBV is known to have complex immunopathological effects. Latent viral proteins encoded by EBV, such as LMP1, a functional homolog of the tumor necrosis factor receptor family (TNF), promote the oncogenic potential of EBV by activating NF-κB signaling (nuclear factor kappa-light-chain-enhancer of activated B cells), which in turn mediates immune evasion and chronic inflammation.^29^ Previous studies revealed that *LMP1* and *LMP2* are expressed in OSCC^30-32^ and NPC^3^, impacting epithelial cell behavior, proliferation, survival, motility, and invasion. A recent study in South India observed a significant prevalence of EBV positivity among HPV-negative OSCC patients and in cases with recurrence, implicating EBV as a marker of worse prognosis in non-HPV OSCC, particularly in patients who smoke and consumed alcohol.^32^ Their findings align with our observation that EBV positivity may be clinically relevant in HPV-negative cases, especially regarding nodal dissemination.

Moreover, we quantified the viral load of EBV in OSCC tumor tissue. Despite not achieving statistical significance, observed trends in the relation between increased EBV viral load and advanced stage and nodal involvement are in line with detected link between EBV positivity and lymphatic dissemination. Our findings suggest that viral copy number may contribute to disease aggressiveness, but the modest sample size (N=39 EBV-positive cases) and wide viral load range likely limited the ability to achieve statistical significance. Moreover, viral detection alone cannot fully explain EBV’s role in cancer, as oncogenic effects also depend on latent protein expression, genetic background, and viral-host immune interactions.

Although the *EBI3* rs428253 variant was not associated with overall survival in the OSCC cohort as a whole, we observed its prognostic significance in EBV-positive tumors, for which the CC genotype predicted worse survival. Bioinformatic analysis confirmed regulatory potential for all analyzed variants, with rs428253 showing the highest functional relevance. Our findings support the hypothesis that viral and host genetic factors might have a combined effect on immune suppression and tumor aggressiveness. However, our findings should be regarded as exploratory and hypothesis-generating. Given the modest number of OSCC patients and small number of CC carriers in our cohort, these findings require validation by larger, independent studies.

*EBI3* rs4740 and rs4905 were also associated with altered OSCC risk under multiple genetic models in our study. The rs4905 A allele was linked to an increased OSCC risk in the dominant model, whereas the AG genotype showed a protective effect in the over-dominant model, highlighting the functional complexity of this locus. Studies investigating the association between *EBI3* gene polymorphisms and cancers are very scarce, and ours is the first to investigate the association between these *EBI3* polymorphisms and OSCC. Prior studies have linked rs4740 to osteosarcoma susceptibility.^22^ However, most of the existing research is predicated on the correlation between *EBI3* polymorphisms and autoimmune or inflammatory disorders.^23,33^

Functionally, *EBI3* encodes a p40 subunit that pairs with p28, p35, or p19 to form IL-27, IL-35, and IL-39, respectively, cytokines with context-specific roles in cancer immunity. IL-27 has both antitumor and pro-tumor activities, and IL-35 has mainly pro-tumor activity.^36,37^ Although p35 and p40 primarily act together as IL-35, each subunit also possesses independent immunoregulatory functions.^38^ EBI3 also interferes with IL-6 trans-signaling and interacts with IFN-γ, IL-4, and IL-10, modulating regulatory T-cells (Tregs) response and immune homeostasis. Moreover, the diversity of EBI3 functions is caused by the lack of a membrane-anchoring domain, which allows EBI3 to function as both an intracellular and secreted immunomodulator.^14^ Autocrine secreted EBI3 promotes T-cell activation and differentiation into Th1, Th17, and Treg cells, as well as B-cell activation and differentiation into plasma cells via the gp130-STAT3 signaling pathway, consequently playing an important role in infection, inflammation, and autoimmune disorders.^33^ Moreover, *EBI3* expression is triggered primarily by the activation of NF-κB in response to various stimuli from TLR ligands and through the T cell receptor.^39^ In other malignancies, high *EBI3* expression has been associated with poor prognosis, lymph node metastasis, and advanced clinical stages in lung,^19^ breast,^17^ gastric,^15^ colorectal,^18^ and nasopharyngeal carcinoma.^21^ Conversely, high *EBI3* expression has been linked to favorable tumor-infiltrating lymphocytes and improved survival in melanoma,^40^ suggesting cancer type-specific effects.

In contrast to EBV, HCMV showed no significant associations with clinicopathological characteristics, survival outcomes, or genetic variants in our HPV-negative OSCC cohort. Despite our negative findings regarding HCMV involvement in HPV-negative OSCC, the role of HCMV in oral carcinogenesis cannot be excluded, as recent reports, though limited, suggest a potential oncoprotective effect in OPSCC and OSCC.^13^

### Mechanistic interpretation based on regulatory context

Bioinformatics analysis revealed that all studied polymorphisms possess regulatory potential, with rs428253 showing the highest regulatory score and overlapping with IRF1 binding sites, suggesting a role in interferon-mediated immune responses, a pathway relevant to viral oncogenesis. This may explain the most pronounced effect of rs428253, specifically in EBV-positive tumors.

Meanwhile, rs4740 and rs4905, despite being in weakly repressed chromatin domains, exhibited strong linkage disequilibrium and disrupted multiple transcription factor binding motifs, supporting their association with tumor stage and protective effects in cancer risk. These regulatory annotations support the hypothesis that genetic variants modulate host immune response to EBV infection, creating the observed genotype-virus-phenotype interactions in OSCC.

### Clinical translation and therapeutic implications

The EBV*-EBI3* axis identified here has immediate translational relevance for HPV-negative OSCC management. This axis represents a regulatory network where EBV infection drives EBI3-mediated immune suppression, creating a tumor-permissive immune microenvironment that influences patient outcomes. Importantly, this axis provides a mechanistic basis for the broader host-virus-tumor interaction framework observed in HPV-negative OSCC, showing how genetic variants modulate viral oncogenic effects to determine tumor behavior and patient prognosis.

This multi-component biomarker system offers several complementary clinical applications. For precision medicine approaches, the EBI3-EBV interaction pattern suggests that EBV-positive patients should undergo additional *EBI3* genotyping to identify those at highest risk (rs428253 CC carriers). This subgroup may benefit from intensified adjuvant therapy or novel immunomodulatory approaches targeting the IL-35/IL-27 pathways. For population-level risk assessment, the protective effects of *EBI3* rs4740 and rs4905 variants in cancer risk suggest these could potentially be incorporated into screening algorithms, particularly for individuals with other oral cancer risk factors. For therapeutic targeting, the axis components represent druggable pathways: EBV through antivirals and *EBI3* through IL-35/IL-27 inhibitors, enabling combination approaches tailored to individual genetic and viral profiles. However, clinical implementation requires prospective validation studies to confirm these associations in larger, diverse cohorts and to establish standardized testing protocols and risk stratification algorithms.

### Scientific contribution and novel insights

This study presented a comprehensive analysis integrating *EBI3* genetic variants with tumor EBV status in OSCC, revealing genotype-virus interactions. Our integration of host and viral biomarkers provides a novel systems-level perspective on the tumor-host-virus triad, uncovering specific genotype-virus interactions such as the prognostic impact of rs428253 exclusively in EBV-positive tumors. The EBV-*EBI3* axis observed here could represent a molecular example of a broader host-virus-gene mechanism in which genetic polymorphisms modulate immune signaling in response to viral infection. This systems-level approach provides a mechanistic framework for understanding tumor heterogeneity in HPV-negative OSCC and establishes a paradigm for investigating similar host-virus-genetic interactions in other virus-associated malignancies.

### Study limitations and future directions

Our study has several limitations that must be addressed. The cohort size was modest, which may reduce the statistical power of subgroup analyses. The control group of healthy individuals was hospital-based, which may not fully represent the general population. This study focused on association analysis; however, these associations are hypothesis-generating and require experimental validation. The lack of functional analysis (such as luciferase reporter assays, expression analysis, or chromatin immunoprecipitation) must be acknowledged as a limitation. Future functional validation in larger studies is needed to confirm predicted transcription factor binding disruptions and potential impact on gene expression. As OSCC is a multifactorial disease, other confounders such as comorbidities or treatment variation could influence outcomes. Additionally, our viral load analysis was based on tissue qPCR and a relative estimate normalized to β-globin, rather than absolute quantitative values. The limited range of viral load values and relatively small number of EBV-positive tumors reduced statistical power. Since EBV viral load was only assessed in tumor tissue, we were unable to compare systemic reactivation between patients and controls. Thus, the presence of EBV DNA in tumors reflects local infection rather than systemic reactivation. Future studies including matched blood/tumor samples and serial viral load monitoring would be valuable to address the hypothesis that EBV contributes to the tumor microenvironment through viral reactivation. Moreover, our EBV detection reflected general LMP region DNA presence rather than isoform-specific expression, such as LMP-1 or LMP-2. Even though EBV status showed clinical associations, viral load evaluation requires larger cohorts and standardized quantification methods to reveal prognostic relevance. These limitations underscore that the present findings are hypothesis-generating, requiring replication in larger cohorts. Further studies are needed to unravel the molecular mechanisms by which these polymorphisms and EBV contribute to OSCC risk or prognosis.

## Conclusion

*EBI3* genetic variants rs4740, rs4905, and rs428253 may be linked to tumor progression, particularly in EBV-positive patients, suggesting that host immunogenetic factors and viral infection may cooperatively drive OSCC aggressiveness. The observed associations between EBV status and *EBI3* genetic variants suggests a possible functional EBV–*EBI3* axis in HPV-negative OSCC. The interplay between EBV-driven inflammation and EBI3-mediated immunomodulation could establish a tumor-permissive immune landscape. These findings support a further larger and functional investigation into the EBV–*EBI3* axis as a potential mechanistic pathway contributing to OSCC progression, particularly in HPV-negative cases.
